# Dyslexia, School‐Connectedness, Depression, and Anxiety During the Transition From Primary to Secondary School

**DOI:** 10.1002/dys.70040

**Published:** 2026-07-13

**Authors:** Robannie A. Sumner, Kate E. Tonta, Adrienne Wilmot, Elizabeth Hill, Mark E. Boyes

**Affiliations:** ^1^ Curtin School of Population Health Curtin University Perth Western Australia Australia; ^2^ Curtin enAble Institute, Faculty of Health Sciences Curtin University Perth Western Australia Australia; ^3^ Centre for Clinical Interventions Perth Western Australia Australia; ^4^ Curtin School of Allied Health Curtin University Perth Western Australia Australia

**Keywords:** dyslexia, mental health, peer relationships, school transition, school‐connectedness

## Abstract

Children with dyslexia are at elevated risk of depression and anxiety. The transition from primary to secondary school may be particularly challenging given increased educational demands. Past research indicates school‐connectedness may be a protective factor across this transition. We tested longitudinal relationships between dyslexia, school‐connectedness, depression, and anxiety over the secondary school transition. Children (*N* = 208, *M*_age_ = 12.16, 58% girls, 61 with dyslexia) and their caregivers (95% mothers) completed standardised measures of school‐connectedness, depression, and anxiety in Year 6, and again in Year 7 after the transition to secondary school. Mediation models tested direct and indirect effects of dyslexia on Year 7 depression and anxiety through domains of school‐connectedness (whole school, peer, friend, and teacher). After adjusting for Year 6 depression, anxiety, and gender, there were no significant direct effects of dyslexia on depression or anxiety in Year 7. However, dyslexia was indirectly associated with both depression and anxiety via school‐ and peer‐connectedness. These findings support the role of school‐connectedness in supporting mental health, and suggest peer‐connectedness might be an important protective factor post‐transition to secondary school. Future studies should explore further change across adolescence and mental health promotion targeting school‐ and peer‐connectedness among children with dyslexia.

## Summary

- Children with dyslexia report lower connection to school and peers relative to children without dyslexia.
- Lower connection to school and peers may partially explain depression and anxiety among children with dyslexia post‐transition to secondary school.
- Whole school programs targeting school‐ and peer‐connectedness could provide neurodiversity‐affirming ways of promoting mental health among children with dyslexia.

## 1 Introduction

Dyslexia is characterised by persistent reading difficulties and impacts approximately 7% of people worldwide, equating to about 2–3 children in every classroom (Yang et al. 2022). Dyslexia is considered a form of neurodivergence, and often co‐occurs with other neurodivergent experiences such as attention, social, and language differences (Adlof and Hogan 2018; Brimo et al. 2021). Dyslexia, and reading challenges more broadly, are associated with a range of psychosocial difficulties, including risk for poorer academic, occupational, and mental health outcomes across the lifespan (Francis et al. 2019; Smart et al. 2017). Specifically, there is comprehensive evidence of the association between dyslexia and depression and anxiety, with moderate, statistically significant associations across studies (Donolato et al. 2022; Francis et al. 2019). Using data from large longitudinal cohorts, reading difficulties predict later depression and anxiety, which suggests dyslexia is a risk factor for these mental health concerns (Jordan and Dyer 2017; McArthur et al. 2022). Consistent with this, when interviewed some (but not all) children and caregivers describe experiencing dyslexia‐related anxiety and stress, particularly within the school environment (Leitão et al. 2017; Wilmot, Pizzey, et al. 2023a, 2023b). While there is robust evidence of the relationship between dyslexia and depression and anxiety, the reasons underpinning these associations remain unclear (Boyes et al. 2016). Exploration of the mechanisms underlying this relationship is needed to inform evidence‐based intervention and prevention for depression and anxiety among children with dyslexia, and the contexts within which they can be best supported (Boyes et al. 2016).

### 1.1 School Experiences and Secondary School Transition

Bronfenbrenner's ecological systems theory conceptualises mental health as a result of interactions between an individual and their multiple, nested environments (Bronfenbrenner 1979). This includes close relationships (e.g., with parents, friends, teachers etc.), contexts (e.g., school, neighbourhood etc.), and wider socio‐political influences (e.g., culture, policies etc.). Applying this framework to dyslexia and mental health, it may be that interactions within the school ecosystem are influential in the relationship between dyslexia and depression and anxiety. Consistent with this, children with dyslexia appear to develop mental health concerns as they enter the school context at the start of primary schooling (Jordan and Dyer 2017; McArthur et al. 2022). The school environment is in turn described by some Australian families as a ‘poor fit’, with few accommodations, overwhelming, embarrassing, and difficult tasks, teachers with poor understanding and training, and a lack of focus on the strengths of children with dyslexia (Leitão et al. 2017; Wilmot, Pizzey, et al. 2023a, 2023b). School‐based factors are also highlighted in quantitative literature, including bullying victimisation, school stress, and poor academic self‐concept which are all associated with dyslexia and mental health concerns (Boyes et al. 2020; McArthur et al. 2020; Wilmot, Hasking, et al. 2023).

Despite evidence of these challenging school experiences, there has been comparatively little examination of school experiences after primary school (Wilmot, Hasking, et al. 2023). This includes over the transition from primary to secondary school (in Australia this transition occurs during the move from Year 6 to Year 7). The transition to secondary school can be challenging for all children but may be particularly stressful for children with dyslexia due to increased academic demands, decreased social support, and increased risk for peer difficulties (Evans et al. 2018; Jindal‐Snape et al. 2020). Indeed, neurodivergent children report more concerns about transition, higher anxiety, higher bullying victimisation, lower social support, and lower school‐connectedness after transition than peers (Cantali 2019; Hebron 2018; Palikara et al. 2026). Further, some neurodivergent children face a lack of teacher awareness and understanding in this environment, which can lead to unmet needs and experiences of shame (Sciberras 2024). However, there have been few studies examining mental health and well‐being over secondary school transition among children with dyslexia. This is notable as, despite common experiences such as increased anxiety, there are also considerable variations in transition experiences between neurodivergent individuals and diagnoses (Palikara et al. 2026; Sideropoulos et al. 2025). Such variation highlights the need for studies examining mental health outcomes and risk and protective factors across transition for children with dyslexia in particular, reflecting their distinct needs and experiences.

In qualitative literature, Australian children with dyslexia and their caregivers describe mixed feelings regarding transition, including concerns for academic demands, wellbeing, and the suitability of support; but also, excitement for the new learning environment (especially when transitioning with supportive friends; Wilmot, Pizzey, et al. 2023a, 2023b). This excitement also marks the transition to secondary school as an important and utile opportunity to create more positive school environments and communities for children with dyslexia, who may have had challenging experiences at school thus far.

### 1.2 School‐Connectedness

School‐connectedness is a multidimensional construct that can be broadly defined as the quality of the relationship between a student and their school environment, including their learning and the people they interact with (e.g., peers and teachers; García‐Moya et al. 2019; Raniti et al. 2022). There is well‐established evidence that strong school‐connectedness protects against adverse mental health outcomes, including moderate metanalytic effect sizes between poor school‐connectedness and depression and anxiety (Allen et al. 2024; Raniti et al. 2022; Yuen and Wu 2024). Interventions addressing school‐connectedness among children are generally effective, indicating it is a modifiable factor (Allen et al. 2022). School‐connectedness has also been named a protective factor for mental health across the transition to secondary school, with stronger school‐connectedness pre‐transition predicting lower depression and anxiety post‐transition (Lester and Cross 2015; Lester et al. 2013; Vaz et al. 2014). Given the challenging school experiences children with dyslexia may be subject to, it is plausible that they may experience lower school‐connectedness (relative to their peers), which may in turn be associated with poorer mental health outcomes. School‐connectedness is therefore a promising avenue for mental health promotion among children with dyslexia, especially across the secondary school transition where increased school‐related stressors and lack of understanding could disconnect children further.

Wilmot et al. (2024) examined school‐connectedness in the context of dyslexia and mental health, using data from Australian caregivers and children in Year 6, with and without dyslexia. Using two mediation models (child‐ and caregiver‐report), they tested whether the associations between dyslexia and internalising (depression and anxiety) or externalising problems (conduct problems) operated indirectly through four domains of school‐connectedness (connection to whole school, peers, friends, and teachers). In both models, dyslexia had no direct effect on internalising nor externalising problems yet had a significant indirect effect on internalising and externalising problems via lower connection to school. Of note, lower school‐connectedness also had a medium to large effect on depression and anxiety in both the child‐ and caregiver‐reported data. However, as the study was cross‐sectional the direction of effects could not be assessed.

### 1.3 The Current Study

We aim to examine the role of school‐connectedness in understanding the relationship between dyslexia and depression and anxiety longitudinally, over the transition from primary to secondary school. Reflecting current recommendations in the literature (i.e., Palikara et al. 2026) we will centre child‐reported data, using caregiver‐reported data to complement results. This paper will directly extend Wilmot et al. (2024) findings by (i) testing the direction of key effects between dyslexia, school‐connectedness and depression and anxiety, and (ii) providing important insight into mental health and school‐connectedness across the secondary school transition. Specifically, we will test the hypothesis that dyslexia is associated with depression and anxiety after secondary school transition both directly and indirectly via domains of school‐connectedness (whole school, peer, friend, and teacher). Findings may inform interventions to address school‐connectedness and mental health during the transition from primary to secondary school.

## 2 Methods

Data for this study were collected as part of a larger study on mental health among children with and without reading difficulties. Participants were recruited through social media, schools, word of mouth, and a service provider for children with learning difficulties in Western Australia. Inclusion criteria were that children lived in Australia and were in Year 6 (final year of primary school) and had a caregiver willing to participate. Participants were followed up in Year 7 (first year of secondary school) to repeat the assessment.

### 2.1 Participants

At timepoint 1 (Year 6), 283 children (*M*_age_ = 11.12 years; 53% girls; 31% with dyslexia) and their caregivers (95% mothers) participated. At timepoint 2 (Year 7), 208 children (*M*_age_ = 12.17 years; retention rate of 73%) and their caregivers provided follow‐up data. Based on guidelines by Fritz and Mackinnon (2007), this study was sufficiently powered to find the expected moderate effects in line with Wilmot et al. (2024) findings. Of these Year 7 children, 92 had received at least one neurodevelopmental diagnosis (44%), with 61 reporting a diagnosis of dyslexia (29%) and 53 a diagnosis of ADHD (25%). In terms of gender, 120 child participants identified as girls (58%), 85 as boys (41%), two responded ‘prefer not to say’, and one responded ‘other’.

### 2.2 Measures

Along with information on sex and neurodevelopmental diagnoses, the following information was collected.

#### 2.2.1 Dyslexia

Caregivers were asked to report whether their child had received a diagnosis of dyslexia. In Australia, dyslexia diagnosis is typically provided by a psychologist and informed by the Diagnostics and Statistics Manual (5th edition, DSM‐5‐TR) criteria for specific learning disorder with difficulty in reading (American Psychiatric Association [APA] 2022; Sadusky et al. 2022). These criteria include (1) persistent difficulties in word recognition, reading fluency, and reading comprehension, (2) academic skills well below the average range in reading despite targeted intervention, (3) difficulties beginning at school‐age, and (4) difficulties not explained by any other condition or adversity (APA 2022).

#### 2.2.2 Depression and Anxiety

The 25‐item youth and caregiver version of the Revised Children's Anxiety and Depression Scale (RCADS‐25) was used to measure depression and anxiety symptoms at both timepoints (Ebesutani et al. 2012, 2017). The RCADS‐25 contains a depression subscale (10 items) and anxiety subscale (15 items). Participants are required to rate how frequently each item (e.g., “I feel sad or empty”) happens to them or their child on a 4‐point scale ranging from “Never” to “Always,” with higher scores indicating higher levels of depression or anxiety. The RCADS‐25 is a recommended depression and anxiety measure based on The Common Measures in Mental Health Science Initiative (Farber et al. 2023). Further, the RCADS‐25 and the 47‐item version from which it was developed have demonstrated sufficient psychometric properties for research, including among neurodivergent samples (Becker et al. 2019; Piqueras et al. 2017). Internal consistency for children who participated in this study at both timepoints was good, with alphas ranging between *α* = 0.83 (depression at timepoint 1) and *α* = 0.88 (anxiety at timepoint 2). Caregiver data also showed high consistency, with alphas ranging between *α* = 0.83 (depression at timepoint 1) and *α* = 0.86 (anxiety at timepoint 1; depression at timepoint 2).

#### 2.2.3 School‐Connectedness

The Hemingway Measure of Adolescent Connectedness (HMAC) was used to assess various domains of *school‐related* connectedness at both timepoints (adolescent version 5.5; Karcher 2011). The HMAC uses an ecological, multidimensional approach, and captures feelings and behaviours related to independent domains of connectedness (Karcher 2011). Four subscales of the HMAC were used to capture school‐related connectedness, including connectedness to the school context and learning (e.g., “I work hard at school”; referred to as “school‐connectedness”), school‐based peers (e.g., “I like working with my classmates”; referred to as “peer‐connectedness”), friends (e.g., “I spend as much time as I can with my friends”; referred to as “friend‐connectedness”), and teachers (e.g., “I usually like my teachers”; referred to as “teacher‐connectedness”). Participants were required to rate their agreement to each statement on a 5‐point scale ranging from “Not at all True” to “Very True,” with higher subscale scores indicating higher connectedness in the relevant domain. The HMAC has demonstrated sufficient psychometric properties for research with children (i.e., *α* > 0.72; Karcher 2011; Too et al. 2022). Internal consistency among the final sample of this study ranged from *α* = 0.77 (teacher‐connectedness; child‐report) to *α* = 0.87 (peer‐connectedness; caregiver‐report) at timepoint 1; and *α* = 0.79 (school‐connectedness; child‐report) to *α* = 0.90 (peer‐connectedness; caregiver‐report) at timepoint 2.

### 2.3 Procedure

Ethics approval was provided by Curtin University's Human Research Ethics Committee. Caregiver consent and child assent were collected electronically via the survey prior to data collection. Children and their caregivers met with a trained research assistant who administered surveys and literacy assessments either in‐person or online through videoconferencing software. The assessment and surveys took approximately one hour. Research assistants remained with participants to answer questions, read the survey aloud (if required), and monitor for distress. Participants were reminded that they could stop or take a break at any time. If clinical levels of depression or anxiety were reported (based on norms and clinical cut‐offs for the RCADS‐25), a registered psychologist on the team contacted caregivers to provide information on available supports (Timepoint 1: *n* = 55; Timepoint 2: *n* = 38). Children received a $15 gift voucher after each assessment to thank them for their participation.

### 2.4 Data Analysis Plan

After data cleaning and screening, child and caregiver participants who participated in Year 7 (post‐transition) were compared to those who were lost to follow‐up on all variables of interest in Year 6 (pre‐transition). Second, descriptive statistics, bivariate correlations, and group differences between participants with and without dyslexia were computed using only participants who took part in both Year 6 and 7. Finally, tests of direct and indirect effects were conducted using the lavaan package (Rosseel 2012) in JASP v. 019 (JASP team 2024). Separate models for child‐reported data and caregiver data were tested. Depression and anxiety in Year 7 were entered as outcome variables, domains of school‐connectedness (whole school, peer, friend [child‐reported only], and teacher) in Year 7 as mediators, and dyslexia diagnosis in Year 6 as the predictor. All models adjusted for gender and baseline depression and anxiety scores. Covariances between all domains of school‐connectedness and between anxiety and depression across timepoints were identified in the model. A bootstrapping procedure (5000 resamples) produced bias‐corrected coefficients and confidence intervals for all direct and indirect effects, as recommended by Biesanz et al. (2010).

## 3 Results

### 3.1 Preliminary Analyses

Based on independent samples *t*‐tests and chi square tests of contingencies (with *α* = 0.05; for categorical variables), there were no statistically significant differences in school‐connectedness, peer‐connectedness, friend‐connectedness, caregiver‐reported teacher‐connectedness, depression, anxiety, dyslexia diagnoses, or other neurodevelopmental diagnoses between participants who were retained (*n* = 208) and those who were lost to Year 7 follow‐up (*n* = 75). Participants who were lost to follow‐up did however have significantly lower scores on child‐reported teacher‐connectedness (*t*[281] = −2.86, *p* = 0.002, two‐tailed, *d* = −0.39) at baseline than those who were retained. Similarly, significantly fewer males participated in Year 7 than expected (*χ* ^2^ [1, *N* = 283] = 8.0, *p* = 0.005, *ϕ* = −0.17), suggesting there was differential attrition by sex.

From this point, only participants who took part in both Year 6 and Year 7 were included in analyses. Nonsignificant Little's MCAR tests indicated data was missing completely at random from both child and caregiver data. Given this and the small amount of missing data per item (< 2% across the dataset), missing values were imputed using expectation maximisation.

### 3.2 Correlations and Group Differences

Disaggregated means for participants with and without dyslexia are reported in Table 1, including note of significant group differences. Correlations are reported in Table 2.

**TABLE 1:** Group means and differences between children with dyslexia and neurotypical peers.

Timepoint	Variable category	Source	Variable	Mean (SD)	Mean (SD)
				Dyslexia (*N* = 61)	No dyslexia (*N* = 147)
Year 6	School‐connectedness	Child	School‐connectedness	19.98 (3.81)**	22.65 (4.07)**
			Peer‐connectedness	20.84 (5.05)*	22.41 (3.98)*
			Friend‐connectedness	24.43 (4.46)	24.37 (4.51)
			Teacher‐connectedness	23.28 (4.22)	23.86 (4.01)
		Caregiver	School‐connectedness	19.92 (4.20)**	23.64 (4.34)**
			Peer‐connectedness	18.59 (3.82)*	19.94 (3.57)*
			Teacher‐connectedness	25.82 (3.50)	25.35 (3.85)
	Mental health outcomes	Child	Depression	9.05 (4.93)*	7.37 (4.61)*
			Anxiety	12.56 (7.51)*	10.75 (6.65)*
		Caregiver	Depression	6.08 (4.20)*	4.69 (4.10)*
			Anxiety	8.18 (6.38)*	6.65 (4.68)*
Year 7	School‐connectedness	Child	School‐connectedness	19.70 (4.15)*	21.33 (4.41)*
			Peer‐connectedness	19.90 (4.57)**	22.31 (4.27)**
			Friend‐connectedness	24.44 (4.44)	24.70 (4.26)
			Teacher‐connectedness	21.90 (4.58)	22.34 (4.62)
		Caregiver	School‐connectedness	19.97 (4.64)**	22.76 (4.63)**
			Peer‐connectedness	18.07 (3.99)*	19.20 (4.64)*
			Teacher‐connectedness	23.74 (4.08)	23.83 (4.20)
	Mental health outcomes	Child	Depression	8.98 (5.20)	7.87 (5.05)
			Anxiety	11.79 (7.72)	10.33 (7.15)
		Caregiver	Depression	6.25 (4.77)	5.25 (4.34)
			Anxiety	7.30 (5.47)*	5.78 (4.34)*

*Note:* *p < 0.05, **p < 0.01; Significant mean differences assessed using independent samples *t*‐tests.

**TABLE 2:** Correlations between variables of interest across year 6 and year 7.

Timepoint	Variable category	Source	Variable	1	2	3	4	5	6	7	8	9	10	11	12
	Demographics	Caregiver	1. Dyslexia	—											
			2. Gender	−0.05	—										
			3. Other NLD	−0.41**	−0.12	—									
Year 6	School‐connectedness	Child	4. School‐connectedness	−0.29**	−0.14	−0.34**	—								
			5. Peer‐connectedness	−0.16*	0.01	−0.23**	0.56**	—							
			6. Friend‐connectedness	0.01	−0.14*	−0.11	0.34**	0.54**	—						
			7. Teacher‐connectedness	−0.07	−0.15*	−0.20**	0.57**	0.34**	0.24**	—					
		Caregiver	8. School‐connectedness	−0.37**	−0.20**	−0.41**	0.62**	0.28**	0.21**	0.29**	—				
			9. Peer‐connectedness	−0.17*	< 0.01	−0.26**	0.35**	0.51**	0.35**	0.15*	0.54**	—			
			10. Teacher‐connectedness	0.06	−0.25**	−0.09	0.36**	0.07	0.13	0.36**	0.49**	0.26**	—		
	Mental health outcomes	Child	11. Depression	0.16*	−0.10	0.21**	−0.56**	−0.47**	−0.21**	−0.24**	−0.34**	−0.37**	−0.17*	—	
			12. Anxiety	0.12	−0.22**	0.12	−0.42**	−0.35**	−0.16*	−0.07	−0.29**	−0.34**	−0.11	0.77**	—
		Caregiver	13. Depression	0.15*	−0.07	0.45**	−0.36**	−0.35**	−0.21**	−0.14*	−0.47**	−0.57**	−0.10	0.42**	0.35**
			14. Anxiety	0.13	−0.22**	0.28**	−0.24**	−0.23**	−0.15*	−0.06	−0.31**	−0.51**	−0.01	0.39**	0.47**
Year 7	School‐connectedness	Child	15. School‐connectedness	−0.17*	−0.09	−0.27**	0.61**	0.38**	0.19**	0.45**	0.42**	0.29**	0.23**	−0.47**	−0.30**
			16. Peer‐connectedness	−0.25**	−0.05	−0.30**	0.37**	0.58**	0.30**	0.21**	0.24**	0.37**	0.05	−0.43**	−0.33**
			17. Friend‐connectedness	−0.03	−0.26**	−0.17*	0.21**	0.25**	0.42**	0.23**	0.11	0.16*	0.11	−0.11	0.04
			18. Teacher‐connectedness	−0.04	−0.22**	−0.26**	0.47**	0.29**	0.16*	0.57**	0.33**	0.11	0.37**	−0.28**	−0.10
		Caregiver	19. School‐connectedness	−0.27**	−0.11	−0.29**	0.51**	0.31**	0.14*	0.34**	0.64**	0.39**	0.31**	−0.34**	−0.27**
			20. Peer‐connectedness	−0.13	0.05	−0.29**	0.20**	0.38**	0.28**	0.14*	0.41**	0.69**	0.20**	−0.34**	−0.32**
			21. Teacher‐connectedness	−0.01	−0.13	−0.16*	0.43**	0.16*	0.04	0.44**	0.44**	0.24**	0.58**	−0.19**	−0.11
	Mental health outcomes	Child	22. Depression	0.10	−0.12	0.22**	−0.28**	−0.32**	−0.18*	−0.06	−0.18**	−0.36**	−0.05	0.59**	0.45**
			23. Anxiety	0.09	−0.26**	0.11	−0.18**	−0.23**	−0.06	−0.07	−0.12	−0.30**	−0.02	0.48**	0.59**
		Caregiver	24. Depression	0.10	−0.12	0.36**	−0.28**	−0.33**	−0.24**	−0.12	−0.40**	−0.52**	−0.12	0.34**	0.28**
			25. Anxiety	0.15*	−0.25**	0.27**	−0.18*	−0.17*	−0.08	−0.01	−0.27**	−0.46**	0.02	0.31**	0.40**

Timepoint	13	14	15	16	17	18	19	20	21	22	23	24	25
													
												
												
Year 6													
												
												
												
												
												
												
												
												
	—												
	0.73**	—											
Year 7	−0.27**	−0.19**	—										
	−0.39**	−0.29**	0.40**	—									
	−0.08	0.03	0.31**	0.34**	—								
	−0.14*	−0.06	0.69**	0.27**	0.31**	—							
	−0.41**	−0.29**	0.69**	0.32**	0.18**	0.52**	—						
	−0.50**	−0.50**	0.35**	0.47**	0.21**	0.19**	0.49**	—					
	−0.17*	−0.14*	0.50**	0.07	0.09	0.53**	0.61**	0.29**	—				
	0.42**	0.36**	−0.48**	−0.43**	−0.19**	−0.18*	−0.34**	−0.42**	−0.11	—			
	0.32**	0.44**	−0.31**	−0.36**	0.01	0.01	−0.25**	−0.40**	−0.05	0.71**	—		
	0.73**	0.59**	−0.31**	−0.29**	−0.11	−0.22**	−0.50**	−0.61**	−0.30**	0.46**	0.35**	—	
	0.58**	0.74**	−0.20**	−0.24**	0.02	−0.01	−0.31**	−0.54**	−0.15*	0.40**	0.49**	0.72**	—

*Note:* *p < 0.05, **p < 0.01. Correlations in which one variable is binary (i.e., dyslexia, gender, other NLD) are point biserial.

Abbreviation: NLD = neurodevelopmental diagnosis (other than dyslexia).

As expected, when comparing children with and without dyslexia, those with dyslexia had significantly higher depression and anxiety in Year 6 in both child‐ and caregiver‐reported data. In Year 7 however, only caregiver‐reported anxiety was significantly higher among children with dyslexia. In both child‐ and caregiver‐reported data, children with dyslexia demonstrated significantly poorer school‐ and peer‐connectedness across both Year 6 and 7, with no significant differences in teacher‐ or friend‐connectedness at either timepoint. Further, all school‐connectedness domains at both timepoints showed significant, negative correlations with at least one mental health outcome.

As predicted, depression and anxiety prior to secondary school transition showed significant, moderate‐to‐large correlations with depression and anxiety after secondary school transition. Similarly, gender showed a small, significant correlation with anxiety before and after secondary school transition, with girls reporting higher anxiety than boys. Given these findings, gender as well as depression and anxiety in Year 6 were entered as covariates on mental health outcomes in both models (child‐ and caregiver‐report).

### 3.3 Tests of Direct and Indirect Effects

**FIGURE 1:**
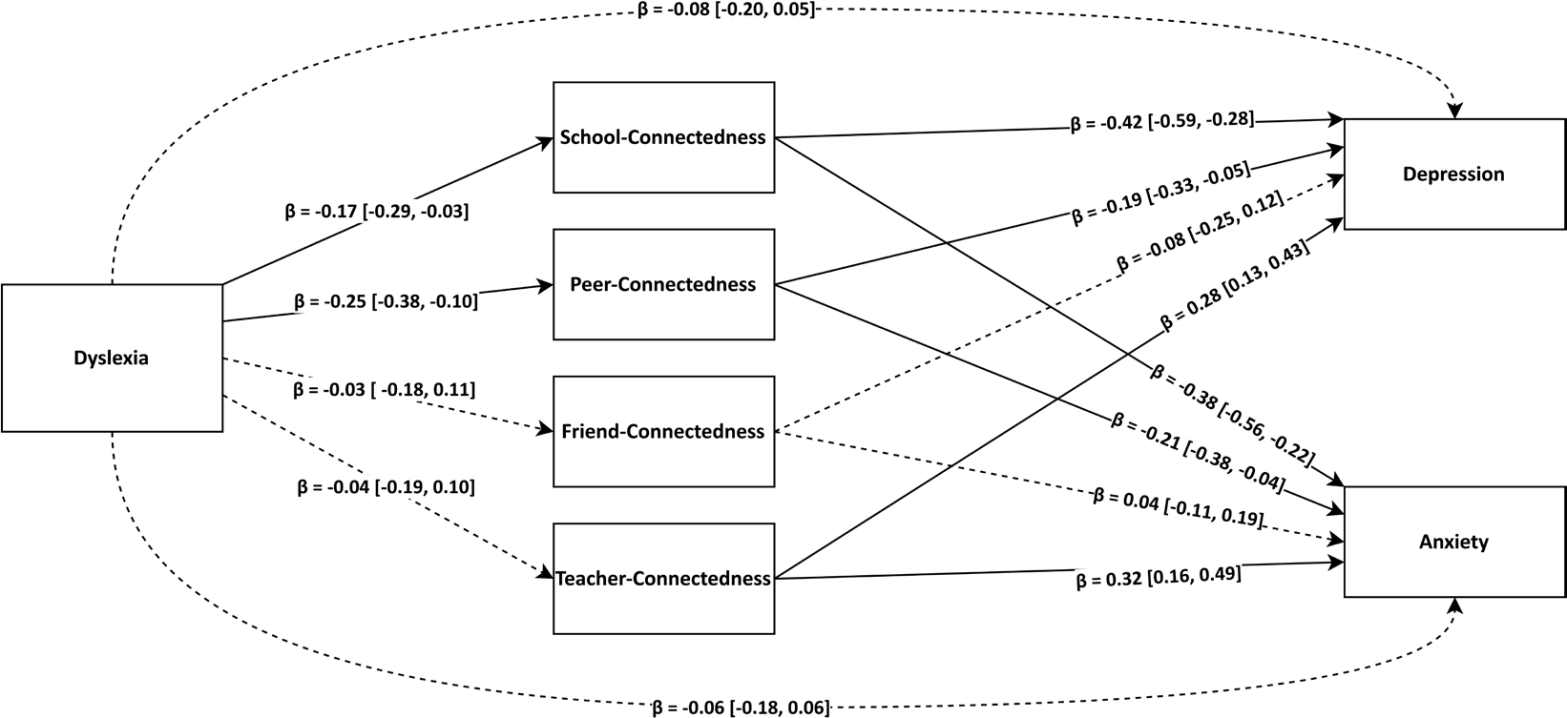
Direct and indirect effects of dyslexia on depression and anxiety after secondary school transition: Child report.

In both the child data and caregiver data, there were no significant direct effects of dyslexia on depression or anxiety in Year 7 (bias‐corrected 95% confidence intervals [CIs] overlapped with 0). However, in child‐reported data, dyslexia had a significant indirect effect on both Year 7 depression and anxiety via school‐connectedness (depression: *β* = 0.072, 95% CI = [0.019, 0.143]; anxiety: *β* = 0.064, 95% CI = [0.017, 0.145]) and peer‐connectedness (depression: *β* = 0.046, 95% CI = [0.011, 0.106]; anxiety: *β* = 0.051, 95% CI = [0.014, 0.121]); see Figure 1.

**FIGURE 2:**
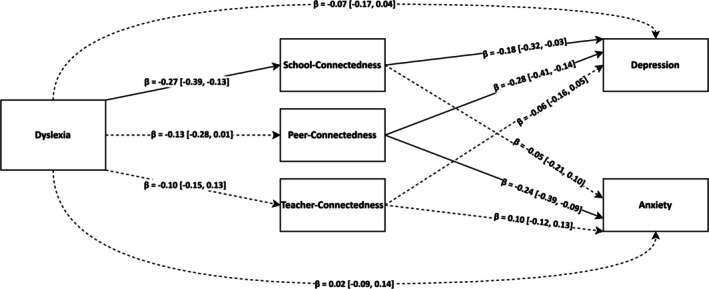
Direct and indirect effects of dyslexia on depression and anxiety after secondary school transition: Caregiver report.

In contrast, the caregiver‐reported data showed dyslexia had significant indirect effects via school‐connectedness on Year 7 depression (*β* = 0.047, 95% CI = [0.010, 0.112]); and via peer‐connectedness on both Year 7 depression (*β* = 0.037, 95% CI = [0.002, 0.096]) and anxiety (*β* = 0.032, 95% CI = [0.001, 0.090]) despite a nonsignificant component pathway (dyslexia to peer‐connectedness); see Figure 2.

## 4 Discussion

The transition from primary to secondary school is a stressful time for all children but may pose a particular risk for children with dyslexia, due to increased academic demands and changes in the learning environment (Evans et al. 2018; Jindal‐Snape et al. 2020). There is preliminary evidence to suggest that school‐connectedness may be a protective factor for the mental health of children with dyslexia during their primary school years (Wilmot et al. 2024) but, to the best of our knowledge, this has not been investigated among students as they transition from primary to secondary school. Using a sample of Australian school students and their caregivers, our aim was to test the hypothesis that dyslexia would be associated with depression and anxiety both directly and indirectly via domains of school‐connectedness across the secondary school transition.

Consistent with previous research on dyslexia and mental health (e.g., Francis et al. 2019), our sample of children with dyslexia and their caregivers reported more symptoms of depression and anxiety prior to secondary school transition relative to peers. However, after the transition only caregiver‐reported anxiety (not child‐report) was significantly higher among children with dyslexia. Indeed, we observed no significant direct effects of dyslexia on depression and anxiety after transition, once adjusting for gender, depression, and anxiety before transition. These findings replicate Wilmot et al.'s (2024) findings in this sample in the final year of primary school, and support the notion that links between dyslexia and mental health concerns are likely explained by other co‐occurring factors.

Previous research indicates that challenging school experiences including peer difficulties (e.g., Weinreich et al. 2023), poor academic self‐concept (e.g., McArthur et al. 2020), and school‐related stress (Alexander‐Passe 2008) may be salient, co‐occurring factors, showing associations with both dyslexia and mental health. Challenges at school are also reflected in qualitative studies, which report experiences defined by struggle, stigmatisation, and Australian school systems that are incongruous with the skills, strengths, and challenges of children with dyslexia (Wilmot, Pizzey, et al. 2023a, 2023b). This highlights the school environment as influential in the wellbeing of children with dyslexia, and thus an important context in which to target mental health promotion efforts. In keeping with this, our findings highlight that school‐connectedness and peer‐connectedness may mediate associations between dyslexia and mental health outcomes.

Specifically, school‐connectedness mediated the associations between dyslexia and child‐reported depression and anxiety; and caregiver‐reported depression. This extends the findings of Wilmot et al. (2024) who tested these associations in a cross‐sectional sample of students at the end of primary school by demonstrating school‐connectedness remains important during the transition to secondary school. This finding is consistent with previous literature which reports lower school‐connectedness among neurodivergent children (e.g., Dimitrellou and Hurry 2019; Hebron 2018) and is likely exacerbated by challenging school experiences and poor environmental “fit” (i.e., Wilmot, Pizzey, et al. 2023a, 2023b). Together, this work highlights that school‐connectedness is associated with depression and anxiety among children with dyslexia, and that these associations may persist across the transition to secondary school. Moreover, our findings provide strong support for the proposed indirect relationships by demonstrating that dyslexia is prospectively associated with lower school‐connectedness, and that these indirect effects exist above what can be accounted for by depression and anxiety in the year prior.

Additionally, our study extends findings from Wilmot et al. (2024) by demonstrating indirect effects of dyslexia on depression and anxiety via peer‐connectedness (child‐ and caregiver‐report) after transition. Consistent with past research which highlights the importance of positive peer relationships for the mental health of children with dyslexia (e.g., Giovagnoli et al. 2020; Leitão et al. 2017) our study found that peer‐connectedness appears especially important in the mental health of children with dyslexia upon beginning secondary school. This may be because the start of secondary school coincides with early adolescence, and thus an increase in the salience and influence of peers socially and neurobiologically (Blum et al. 2022; Laursen and Veenstra 2021). Indeed, peers and “fitting in” among them are consistently reported as central to concerns about transition, including among neurodivergent children (Lester and Cross 2015; Scanlon et al. 2016; Waters et al. 2014). However, children with dyslexia describe experiences of “feeling different” and difficulty “fitting in” which may negatively affect peer‐connectedness, especially when “fitting in” is becoming highly valued developmentally (Eissa 2010; Leitão et al. 2017). Children with dyslexia may also be at increased risk for bullying involvement and social skill challenges (e.g., Kouvava et al. 2025; Weinreich et al. 2023), which may further hinder connection to peers. Consistent with this, children with dyslexia and their caregivers in this study reported poorer peer‐connectedness than peers both before and after the transition to secondary school. The link between peer relationships and mental health in adolescence is well established, with supportive peer relationships being associated with fewer symptoms of depression and anxiety (Alsarrani et al. 2022). Similarly, robust peer support and strong friendships appear central to socioemotional wellbeing and feelings of safety across the transition to secondary school, where primary school peer relationships may be disrupted and a new peer group is introduced (e.g., Heinsch et al. 2020; Waters et al. 2014). Peer‐connectedness and friendships therefore appear vital to facilitating positive transition experiences and may also buffer against the school‐related challenges many children with dyslexia face. As such, peer‐connectedness emerges as another target for future mental health promotion among children with dyslexia, especially over the secondary school transition. Future research may therefore seek to extend upon our findings by looking at the role of peer and social relationships more broadly, including experiences of bullying involvement.

Although the moderate correlations between child‐ and caregiver‐reported depression and anxiety in this sample are to be expected (i.e., De Los Reyes et al. 2015) it is notable that there were consistent patterns of findings across both child‐ and caregiver‐report regarding the relationships between dyslexia, school‐connectedness, and depression and anxiety. This provides robust support for school‐ and peer‐connectedness as potential factors linking dyslexia and depression and anxiety. Further, it suggests that caregivers have some insight into their child's school‐related connectedness with moderate‐to‐strong correlations across variables in our sample. In the context of dyslexia, mothers have described witnessing their child endure school‐related stress and often comment on a lack of support or understanding in the school environment (e.g., Wilmot, Pizzey, et al. 2023b). These challenges can also contribute to greater parental stress and exhaustion among caregivers who engage in ongoing advocacy, support seeking, and navigating their child's mental health and school challenges (Carotenuto et al. 2017; Wilmot, Pizzey, et al. 2023a, 2023b). Such reciprocal interactions between the child, the home, and the school environment are consistent with ecological systems theory (Bronfenbrenner 1979) and highlight the importance of parent‐school connections for promoting family wellbeing (Shilshtein et al. 2025; Smith et al. 2019). Future studies should examine caregiver connections to school and how they may be associated with both child and caregiver mental health outcomes.

### 4.1 Implications

From a theoretical perspective, consistent with ecological systems theory (Bronfenbrenner 1979), our findings highlight that environmental factors are important considerations for understanding mental health in the context of dyslexia. Specifically, this growing evidence for the importance of the school context in mental health for children with dyslexia is well‐aligned with the neurodiversity paradigm. The neurodiversity paradigm (Blume 1998; Singer 1998) is underpinned by the belief that all neurological variations between people (including dyslexia) are equally valid and valuable and thus should not be considered “disordered” nor “deficit” (Pellicano and den Houting 2022). Understanding mental health through this lens encourages research to consider disabling or “othering” community‐ and environment‐level factors, and the “fit” of the contexts neurodivergent people are situated in (Dwyer 2022; Rappolt‐Schlichtmann et al. 2018). Therefore, drawing on relevant aspects of both ecological systems theory and the neurodiversity paradigm, future research could further examine the “fit” of school and other contexts (e.g., community groups or sports clubs, support settings, and work) for individuals with dyslexia.

Practically, this study supports and extends the findings from Wilmot et al. (2024) that school‐connectedness is a plausible mediating factor in the relationship between dyslexia and internalising problems. Distinctly, however, we found that peer‐connectedness may also be important in this relationship across the secondary school transition. Due to the longitudinal design and use of both child‐ and caregiver‐report, findings provide strong, reliable support for school‐ and peer‐connectedness as mechanisms potentially linking dyslexia and depression and anxiety. The robust links between dyslexia, school‐connectedness, and depression and anxiety before and after secondary school transition contribute to growing arguments for school‐based programs promoting mental health by improving school‐connectedness. Relevant to the context of this study, programs targeting school‐connectedness and mental health have been successful in addressing depression among children over the secondary school transition (Blossom et al. 2020) and anxiety among children who are neurodivergent (Shochet et al. 2022). Such programs also align with neurodiversity affirming perspectives as they are community‐level, have a strengths‐based focus, and minimise “othering” (and therefore further disconnect) by including all students, rather than removing students for intervention (Allen et al. 2022; Dwyer 2022). Further, most have a focus on improving school‐based interactions and relationships and thus would target peer‐connectedness simultaneously. These programs, therefore, seem appropriate to adapt to the context of dyslexia, school‐ and peer‐connectedness, mental health, and secondary school transition experiences. Though such programs would likely promote mental health for all children over transition (Lester et al. 2013), they are especially important for children with lower connection to school and peers, thus potentially closing the gap in school‐connectedness, peer‐connectedness, and mental health outcomes for children with dyslexia. Facilitating connections between peers with shared experiences may also help children with dyslexia who have felt othered or struggled to ‘fit in’. For example, neurodivergent student‐led peer support groups aimed at improving feelings of connection, wellbeing, and inclusion at school for children who are neurodivergent (i.e., Crompton et al. 2024; Fotheringham et al. 2023) may be particularly applicable to children with dyslexia with further study on outcomes and efficacy.

### 4.2 Limitations

Findings of our study should be interpreted with several limitations in mind. First, conclusions regarding the trajectory of school‐connectedness and depression and anxiety over the secondary school transition are limited, as data was only collected at one point before and after transition. Future studies should consider using more timepoints or employing designs such as ecological momentary assessment (Shiffman et al. 2008), which could provide a more comprehensive understanding of these dynamic relationships. Further, given evidence that school‐connectedness tends to decline across the secondary school years (i.e., Gillen‐ O'Neel and Fuligni 2013; Sani et al. 2026), children with dyslexia may be particularly at‐risk for mental health concerns in upper secondary school. Future studies should therefore examine school‐connectedness and mental health among children with dyslexia into upper secondary school years.

Although the inclusion of both child‐ and caregiver‐report is a strength of this study, the overrepresentation of mothers in our sample and the lack of other informant perspectives limits our findings. The disproportionate prevalence of mothers is consistent with the literature on child mental health broadly (Parent et al. 2017), and dyslexia and mental health more specifically (Wilmot, Hasking, et al. 2023). This speaks to the need for future research with diverse informants (e.g., fathers, grandparents) to better understand interactions between caregiver perspectives and reports of child mental health and school experiences. Further, perspectives from school‐based peers and teachers may provide insight into how school‐connectedness is experienced and enacted with children with dyslexia. Finally, as participants lost to follow‐up had significantly lower child‐reported teacher‐connectedness, it is possible that the observed effects regarding teacher relationships are underestimated. Boys were additionally lost to follow‐up at a higher rate than girls and thus are under‐represented. Future studies could focus on ensuring that the experiences of boys, and children most disconnected from teachers, are captured.

## 5 Conclusion

Our findings highlight school‐ and peer‐connectedness as possible mechanisms that may help explain the association between dyslexia and depression and anxiety during the transition from primary to secondary school. Importantly, dyslexia was not directly linked to depression or anxiety; rather, dyslexia was associated with lower school‐ and peer‐connectedness, which in turn was associated with higher levels of depression and anxiety. These findings are in keeping with neurodiversity affirming perspectives and suggest the need for school‐based programs and policies to support mental health by targeting the school‐ and peer‐connectedness of children with dyslexia as they transition from primary to secondary school.

## Funding

Mark Boyes was supported by the National Health and Medical Research Council (NHMRC), Australia (Investigator Grant, 1173043) and the Stan Perron Charitable Foundation (People Grant, 202405). Elizabeth Hill was supported by the Stan Perron Charitable Foundation (People Grant, 202552).

## Ethics Statement

This study was approved by Curtin University's Human Resource Ethics Committee (HRE2020‐0168). Informed consent was obtained from adult participants and informed assent from child participants.

## Conflicts of Interest

Mark Boyes is a member of the *Dyslexia* Editorial Board. No other authors have conflicts to declare.

## Data Availability

The data that support the findings of this study are available from the corresponding author upon reasonable request.
